# Multiplex PCR assay for the rapid detection of Klebsiella pneumoniae pathotypes

**DOI:** 10.1099/jmm.0.002090

**Published:** 2025-10-31

**Authors:** Sanika Mahesh Kulkarni, Jobin John Jacob, T. Praveen, V. Aravind, R. Subbulakshmi, S. Preethi, Binesh Lal, Karthik Gunasekaran, Abi Manesh, Shraddha M. Karve, J. Sudarsana, Sanjay Bhattacharya, Anand Shah, Savitha Nagaraj, Priyadarshini Padaki, S. Jayakumar, Renu Mathew, S.M. Rudresh, Shariqa Qureshi, S. Nivedhana, Geethu Joe, Ekadashi Rajni, Kamini Walia, Balaji Veeraraghavan

**Affiliations:** 1 Department of Clinical Microbiology, Christian Medical College, Vellore, India; 2 The Tamil Nadu Dr MGR Medical University, Guindy, Chennai, India; 3 Department of Medicine, Unit V, Christian Medical College and Hospital, Vellore, India; 4 Department of Infectious Diseases, Christian Medical College and Hospital, Vellore, India; 5 Trivedi School of Biology & Koita Centre for Digital Health, Ashoka University, Sonipat, India; 6 Baby Memorial Hospital, Kozhikode, India; 7 Tata Medical Center, Kolkata, India; 8 Zydus Hospitals, Ahmedabad, India; 9 St. John’s Medical College Hospital, Bengaluru, India; 10 Saveetha Medical College, Chennai, India; 11 Believers Church Medical College Hospital, Thiruvalla, Kerala, India; 12 Chacha Nehru Bal Chikitsalaya, New Delhi, India; 13 Rainbow Children's Hospital, Chennai, India; 14 Jupiter Hospital, Pune, India; 15 Mahatma Gandhi Medical College, Jaipur, Rajasthan, India; 16 Division of Epidemiology and Communicable Diseases, Indian Council of Medical Research, New Delhi, India

**Keywords:** carbapenem resistance, convergence, hypervirulence, *Klebsiella pneumoniae*, multiplex assay

## Abstract

**Introduction.** *Klebsiella pneumoniae* (Kp) is a major cause of nosocomial infections, with its evolving pathotypes including multidrug-resistant, hypervirulent (hvKp) and convergent strains posing significant diagnostic and treatment challenges due to combined antimicrobial resistance and virulence.

**Gap Statement.** While there is a pressing requirement for thorough detection of Kp pathotypes, current assays in resource-limited environments are unable to effectively focus on essential carbapenemase and hypervirulence genes with the necessary reliability and precision.

**Aim.** To develop and validate a multiplex PCR (m-PCR) assay capable of simultaneously detecting Kp isolates including those carrying partial or full virulence markers, alongside antimicrobial resistance.

**Methodology.** In this study, an m-PCR assay was designed and optimized for the simultaneous detection of key biomarkers associated with hypervirulent (*rmpA*, *rmpA2*, *iucA*, *peg344* and *iroB*), carbapenem-resistant (*bla*_NDM_, *bla*_OXA-48-like_ and *bla*_KPC_) and convergent Kp pathotypes in clinical isolates. The assay was evaluated on clinical isolates and validated against whole-genome sequencing (WGS) data for accuracy, specificity and sensitivity.

**Results.** The developed m-PCR assay exhibited 100% specificity when compared to WGS data, successfully detecting all target genes without cross-amplification in ATCC control strains. The assay demonstrated high sensitivity, efficiently amplifying bacterial genomes from minimal DNA input as low as 1 ng µl^−1^. Additionally, validation through sequencing confirmed the accuracy of detected amplicons.

**Conclusion.** This m-PCR assay offers a rapid, sensitive and specific diagnostic tool for differentiating Kp pathotypes in clinical settings, aiding in timely intervention and improved infection control measures.

**Impact Statement**

This study presents a multiplex PCR assay for the rapid and accurate detection of *Klebsiella pneumoniae* pathotypes, addressing the challenge of defining hypervirulence due to the diverse and evolving set of markers. By analysing genomic data from public databases, the most well-established virulence and resistance markers were carefully selected to enable comprehensive strain differentiation. The assay simultaneously detects hypervirulent, carbapenem-resistant and classical strains, offering a faster and more targeted alternative to conventional methods. Additionally, its adaptable design allows for modifications based on regional strain variations, making it a valuable tool for both clinical and epidemiological applications. This approach enhances infection control efforts and antimicrobial stewardship by enabling timely and precise pathogen identification, particularly in resource-limited settings. By bridging genomic insights with practical diagnostics, this assay provides a cost-effective, scalable solution to monitor and manage high-risk *K. pneumoniae* infections more effectively.

## Introduction

*Klebsiella pneumoniae* (Kp), a Gram-negative, non-motile bacterium belonging to the order *Enterobacterales*, is known for its rapid dissemination, persistence and phenotypic diversity [1,2]. In hospital settings, Kp poses a significant threat, particularly to critically ill patients in intensive care units, immunocompromised individuals, the elderly and neonates [3,4]. It is a frequent cause of severe infections, including pneumonia, urinary tract infections, sepsis, wound infections and meningitis. Clinically, Kp is classified into two primary pathotypes: classical (cKp), which is responsible for nosocomial infections, and hypervirulent (hvKp), which is linked to community-acquired infections. cKp is further evolved into multidrug-resistant (MDR-Kp) or carbapenem-resistant (CR-Kp) strains based on their antimicrobial resistance (AMR) profiles [5,8].

The prevalence of MDR-Kp in clinical settings has risen dramatically in recent decades. Studies indicate that approximately one-third of hospital-acquired Gram-negative infections are caused by Kp [9]. In India, surveillance data from a multi-hospital network revealed that Kp accounts for 18% of bloodstream infections, with 57% of isolates exhibiting carbapenem resistance [10]. Treating these infections is particularly challenging due to their resistance to most antibiotics, including last-line therapies. Consequently, the World Health Organization has classified CRKp and extended-spectrum *β*-lactamase (ESBL)-producing strains as critical-priority pathogens [11]. The frequent acquisition of virulence factors, metal resistance and other horizontally transferred genetic elements further complicates the management of Kp infections [12,13].

Beyond nosocomial settings, hvKp has emerged as a significant community-acquired pathogen over the past two decades. Historically, hvKp strains exhibited a hypermucoviscous (hmv) phenotype, which was qualitatively assessed using the ‘string test’ [14]. This phenotype, along with enhanced virulence, stems from the overproduction of capsular polysaccharides and siderophores, driven by genes on the virulence plasmid (pLVPK), such as *rmpADC*, *rmpA2*, *iroBCDN* and *iucABCDiutA* [15]. In recent years, hvKp has evolved into MDR-hvKp by acquiring MDR plasmids. Conversely, MDR-Kp strains have integrated virulence plasmids, giving rise to convergent clones that exhibit both resistance and hypervirulence [16]. These strains are most commonly reported in China, Southeast Asia and East Asia, with increasing cases documented in Europe, the USA and other Western countries [15,17]. In India, a study from a tertiary care hospital identified 8% of isolates as belonging to this convergent pathotype [18]. The emergence of CR-hvKp, harbouring resistance genes such as *bla*_KPC_, *bla*_NDM_ and *bla*_OXA-48-like_, has severely limited treatment options, often resulting in untreatable infections. The rising prevalence of MDR-hvKp in hospital settings is a pressing public health concern [19,22]. Differentiating Kp pathotypes is thus essential for assessing infection severity and devising effective treatment strategies.

Traditionally, hvKp identification relied on a positive string test (string >5 mm) and susceptibility to commonly used antibiotics. However, most convergent clones lack hmv and test negative in the string test, limiting their reliability as a specific marker for hvKp [23,24]. Genetic determinants of hypervirulence, such as aerobactin (*iucA*), salmochelin (*iroB*), metabolic transporter (*peg-344*) and regulator of mucoid phenotype (*rmpA*/*rmpA2*), have emerged as more robust biomarkers for PCR-based detection [25,26]. Not all hvKp strains carry the same set of virulence genes, and frameshift mutations in *rmpA/rmpA2* can reduce the sensitivity of PCR assays [26,28]. For this study, hvKp was defined as isolates carrying all five canonical virulence genes: *rmpA*, *rmpA2*, *iucA*, *iroB* and *peg-344*, which are typically co-located on the pLVPK-like virulence plasmid to ensure accurate identification of truly convergent clones [23,29]. Isolates carrying only a subset of these markers (e.g. *iucA*, *rmpA* and *rmpA2*) are referred to as ‘virulence marker–positive cKp’ rather than hvKp. Given the urgent need for a rapid, comprehensive molecular tool to identify and monitor convergent strains carrying both resistance and virulence determinants, we evaluated multiple genotypic biomarkers to distinguish all three Kp pathotypes: CRKp, hvKp and CR-hvKp. Using these biomarkers, this study developed a multiplex PCR (m-PCR) assay to accurately detect carbapenem-resistant hvKp and inform tailored treatment strategies for affected patients.

## Methods

### Genome sequence data retrieval

We have leveraged whole-genome sequencing (WGS) data available at NCBI pathogen detection (https://www.ncbi.nlm.nih.gov/pathogens/) to identify carbapenem-resistant hvKp genomes. This database integrates genome sequences and annotates AMR and virulence genes present in bacterial genomes. Among the 52,502 assembled genomes of Kp available in the database (dated 3 July 2023), we filtered and downloaded genomes with specific virulence markers (*rmpA/rmpA2/iucA/iroB/peg-344*) in NCBI pathogen detection and screened for 12,846 genomes (Table S3, available in Supplementary Material 1). In this study, hvKp was defined strictly as isolates carrying all five canonical markers (*rmpA*, *rmpA2*, *iucA*, *iroB* and *peg-344*). Subsequently, genomes carrying carbapenemase genes such as *bla*_NDM_ and/or *bla*_OXA-48_ family, specific to Indian clinical contexts, were selected using various filter options within the database (https://www.ncbi.nlm.nih.gov/pathogens/refgene/). MLST, virulence genes, resistance and virulence scores of genome assemblies were examined using Kleborate v2.4.1 (https://github.com/klebgenomics/Kleborate/releases/tag/v2.4.1) and BLASTn against reference genes downloaded from the Institut Pasteur database (https://bigsdb.pasteur.fr/cgi-bin/bigsdb/bigsdb.pl?db=pubmlst_klebsiella_seqdef&page=downloadAlleles).

### Primer design

For screening carbapenem-resistant hvKp, the coding regions of genes associated with hypervirulence (*rmpA*, *rmpA2*, *iucA*, *peg3-44* and *iroB*) were extracted from the genome assemblies (*n*=8177) through BLASTn searches locally, employing a sequence identity threshold of >80% and a coverage threshold of >80%. All reported *bla*_NDM_ and *bla*_oxa-48-like_ variant sequences in the NCBI reference gene catalogue (https://www.ncbi.nlm.nih.gov/pathogens/refgene/) were downloaded. Subsequently, the resulting FASTA files were aligned to reference sequences using the MAFFT program (https://github.com/GSLBiotech/mafft). The m-PCR primers were designed for the primary assay, targeting conserved regions of *rmpA*, *rmpA2*, *iucA*, *bla*_NDM_ and *bla*_OXA-48-like_. Additional primers were developed/included for *peg-344*, *iroB*, *bla*_KPC_ and *bla*_CTX-M_ allowing for flexibility in panel customization. Primer design was performed using the Thermo Fisher OligoPerfect Primer Designer (https://apps.thermofisher.com/apps/oligoperfect/). Selection criteria included a G+C content close to 50 mol%, a ΔTm of <2 °C and a length of 18–22 bases. Before empirical testing, these primers were initially evaluated *in silico* against all target and non-target sequences using NCBI primer-blast (https://www.ncbi.nlm.nih.gov/tools/primer-blast/) and *in silico* PCR (https://insilico.ehu.es/PCR/). Specific primers and assays that target *bla*_KPC_ and *bla*_CTX-M_ were adapted from previously published data [30,31]. All oligonucleotide primer sequences used in this study are listed in Table 1 and were synthesized by Integrated DNA Technologies Pvt Ltd, Singapore. Reference standard strains, as well as clinical isolates, were used for primer optimization and *in vitro* testing.

**Table 1.** Oligonucleotide primers used in this study

Target (phenotype)	Gene target	Sequence	Product size	**Concentration** **(µM)**
Hypervirulence	***rmpA***	CTAAAGCAGTTAACTGG	530 bp	1.2
		CATCTTTCATCAACCATTT		
	***rmpA2***	AAGAGTATTGGTTGATAGCCGGA	470 bp	1.2
		AGGTATTTGATGTGCACCATTTT		
	***iucA***	TGGTTAACTCCACTTTTGCCGT	170 bp	1.2
		ACCTTTTACGTTCCAGTACG		
Carbapenemase producing	***bla*_OXA-48-like_**	GGCGTAGTTGTGCTCTGGAA	630 bp	1.2
		TCTTTTGTGATGGCTTGGCG		
	***bla*_NDM_**	TTTGATCGTCAGGGATGGCG	401 bp	1.2
		TGATCAGGCAGCCACCAAAA		
**Target (phenotype)**	**Gene target**	**Sequence**	**Product size**	**Concentration** **(µM)**
Additional genes (hypervirulence)	***peg-344***	TGGGGATAAGTGTGATAAGT	313 bp	1.2
		TTACCGTGTTTTATAGCTGG		
	***iroB***	ATCTACCCTCCGCTCGGAGT	219 bp	1.2
		GTCGTTTTCAAGAATGCTCA		
Carbapenemase producing [30]	***bla*_KPC_**	CGTCTAGTTCTGCTGTCTTG	798 bp	100
		CTTGTCATCCTTGTTAGGCG		
ESBL producer [31]	***bla***_**CTX-M**_	SCNATGTGCAGYACCAGTAARG	560 bp	1.2
		CCGCRATATGRTTGGTGGTG		

### Characterization of bacterial isolates

Kp strains (*n=150*) used in this study were clinical isolates collected between 2022 and 2023 at the Department of Clinical Microbiology, Christian Medical College, Vellore (*n*=47), and from multiple centres across India, including Baby Memorial Hospital, Kozhikode (*n*=76); Zydus Hospital, Ahmedabad (*n*=10); Saveetha Medical College, Chennai (*n*=5); St. John’s Medical College, Bengaluru (*n*=5); BCMCH, Thiruvalla (*n*=3); and TATA Medical Center, Kolkata (*n*=4) Supplementary Material 1 (Table S4). These isolates were sub-cultured on MacConkey agar at recommended culture conditions. Isolates were identified and confirmed as Kp by biochemical tests and MALDI TOF MS (VITEK^®^ MS, bioMérieux). Screening for the hmv phenotype was conducted via the string test, following the described semi-quantitative method [1]. A positive string test was defined by the formation of a mucoid string exceeding 5 mm in length upon contact with an inoculation loop.

### Antimicrobial susceptibility testing

Antimicrobial susceptibility testing was performed by the Kirby–Bauer disc diffusion method according to CLSI 2022 and 2023 guidelines [32,33]. The antimicrobial agents tested included meropenem (10 µg) and ertapenem (10 µg). Controls utilized for the testing included *Escherichia coli* ATCC 25922, *Enterococcus faecium* ATCC 29212 and *Pseudomonas aeruginosa* ATCC 27853.

### DNA extraction and WGS

The study isolates (*n=150*) were cultured in LB broth (Luria Bertani Broth) at 37 °C. Total genomic DNA was extracted from pelleted cells using the Wizard DNA purification kit (Promega, WI, USA). The concentration of extracted DNA was determined using NanoDrop One spectrophotometry (Thermo Fisher Scientific, MA, USA) and Qubit 3.0 fluorometry (Life Technologies, CA, USA), and the samples were stored at −20 °C until further analysis.

A sequencing library was prepared using the Nextra DNA Flex library preparation kit (Illumina, San Diego, CA). Initially, genomic DNA (1 µg) was fragmented, and adapters were ligated to the ends. The ligated DNA libraries underwent size selection, and the resulting products were PCR amplified with index primers following the manufacturer’s instructions. Subsequently, the paired-end library was sequenced on a NovaSeq 6000 platform (Illumina, USA) at Unipath Specialty Laboratory Limited, Ahmedabad, India, generating 2×150 bp reads. Sequencing reads with a PHRED quality score below 20 were discarded, and adapters were trimmed using cutadapt v1.8.1. Quality assessment was performed using MultiQC (https://github.com/ewels/MultiQC).

### Comparative genome analysis

The resulting high-quality reads were then subjected to assembly using Unicycler (https://github.com/rrwick/Unicycler). MLST, virulence genes, resistance and virulence scores were examined using Kleborate (https://github.com/klebgenomics/Kleborate/releases/tag/v2.4.1). The assembled *Klebsiella* genomes were typed using the Ridom SeqSphere+ platform based on the core genome MLST (cgMLST) scheme for *Klebsiella pneumoniae/variicola/quasipneumoniae* available at https://www.cgmlst.org/ncs/schema/Kpneumoniae2267/. A total of 2,358 loci were selected, and alleles were called using the chewBBACA algorithm (https://github.com/B-UMMI/chewBBACA). Minimum spanning trees (MSTs) were constructed and visualized using GrapeTree (https://achtman-lab.github.io/GrapeTree/MSTree_holder.html) based on the cgMLST. Tree nodes were positioned through dynamic rendering, and node style was adjusted by fine-tuning the node size and kurtosis. Nodes were coloured by the pathotype of the isolates, and node sizes were drawn proportionally to the number of isolates.

### Testing and optimization of m-PCR assay

#### m-PCR primer optimization

To optimize the m-PCR assay, each set of primers was individually tested in monoplex PCR reactions to amplify specific gene targets. Initially, combinations of two genes were tested, and the most efficient pair was selected for the initial double PCR. Subsequently, additional primer pairs were sequentially incorporated to form triple PCR reactions. This iterative process was repeated until the final m-PCR assay was successfully developed. The core targets of the assay included *rmpA*, *rmpA2*, *iucA*, *bla*_OXA-48-like_ and *bla*_NDM_, while *iroB*, *peg-344*, *bla*_KPC_ and *bla*_CTX-M_ (included to enable detection of ESBL-producing strains) were standardized and can be incorporated optionally based on specific requirements. The *bla*_CTX-M_ primer set was adapted from Lartigue *et al.* [31], retaining the reverse primer and modifying the forward primer as mentioned in Table 1.

#### m-PCR cyclic condition optimization

The PCR reaction was prepared using the Qiagen Multiplex PCR kit, comprising HotStarTaq DNA polymerase, Multiplex PCR Buffer and a Q-Solution. Each reaction was set up in a 20 µl volume, containing 10 µl of mastermix, 2 µl of Q-Solution and 6 µl of pooled primers (with 2 µl of each forward and reverse primer for 5 genes combined to make a total of 20 µl, to which 80 µl of nuclease-free water was added). Additionally, 2 µl of DNA template was added to each reaction. The PCR amplification was carried out using a Veriti thermal cycler (Applied Biosystems Inc., Foster City, CA). Annealing temperatures ranging from 46 °C to 56 °C were tested to optimize the amplification conditions. Subsequently, 5 µl of the reaction mixture was loaded onto a 2% agarose gel and electrophoresed at 130 V for 45 min to visualize the resulting amplicons.

### PCR specificity and sensitivity

The specificity of the m-PCR assay was evaluated using 150 clinical isolates of Kp and 12 bacterial strains, including reference strains of *Klebsiella quasipneumoniae **ATCC 700603***, Kp ***ATCC BAA-1705*** and ***BAA-1706***, *Escherichia coli **ATCC 35218***, *Pseudomonas aeruginosa **ATCC 27853***, *Acinetobacter baumannii **ATCC 19606***, *Staphylococcus aureus **ATCC 43300*** and *Streptococcus pneumoniae **ATCC 17815***, as well as strains of *Salmonella* Typhi, *Shigella* sp., *Morganella morganii*, *Serratia marcescens* and *Proteus mirabilis* from IHMA. Genomic DNA extraction was carried out following the procedure outlined in the section ‘DNA extraction and WGS’, and PCR amplification was performed as described in the section ‘m-PCR cyclic condition optimization’.

A tenfold serial dilution procedure was adapted to assess the sensitivity of the m-PCR assay. Initially, the positive control strain B1644 (accession ID: GCA_047922575.1) was cultured on MacConkey agar, and a single colony was inoculated into Mueller–Hinton Broth, followed by adjustment to 0.5 McFarland standard. The suspension was serially diluted in sterile saline from 10^−1^ to 10^−8^, and 10 µl from each dilution was plated on MacConkey agar to determine the c.f.u. per millilitre after 24 h incubation at 37 °C. Additionally, 200 µl from each dilution was used for DNA extraction, followed by m-PCR amplification to detect target genes. The experiment, performed in duplicate, determined sensitivity by consistently identifying the lowest dilution (c.f.u. per millilitre).

These DNA samples served as templates for PCR amplification. Further, the DNA was serially diluted to achieve concentrations ranging approximately from 50 ng l^−1^ to 70 pg l^−1^. Subsequently, 2 µl of each dilution was employed as a template to evaluate the sensitivity.

## Results

### Screening of gene targets

A total of 12,846 genomes carrying at least 1 hypervirulence-associated marker (*iucA*, *rmpA*, *rmpA2*, *iroB* and *peg-344*) were identified through a comprehensive screening of the global collection in the NCBI Pathogen Detection Database Supplementary Material 1 (Table S3) and were analysed using Kleborate. However, in line with current consensus definitions, only 2,641 genomes that harboured all 5 core hvKp markers (*iucA*, *rmpA*, *rmpA2*, *iroB* and *peg-344*) were classified as hvKp. Among these, 576 (21.8%) co-harboured carbapenemase genes, representing CR-hvKp. Notably, 1,790 of these hypervirulent isolates lacked both ESBL and carbapenemase genes. Despite the absence of carbapenemase and traditional ESBL genes in a subset of isolates, *bla*_CTX-M_ was identified in 60.38% (7,757 out of 12,846) genomes (indicating a high underlying ESBL burden in the global dataset). In the virulence marker-positive cKp group, the *iucA* gene was the most prevalent, detected in 96.32% (12,374 out of 12,846) of genomes, followed by *peg-344* in 73.9% (9,495 out of 12,846), *rmpA2* in 70% (8,992 out of 12,846), *rmpADC* in 48.25% (6,199 out of 12,846) and *iroB* in 30.83% (3,961 out of 12,846). Additionally, 68.72% (8,828 out of 12,846) of the genomes carried carbapenemase genes. Among these, *bla*_KPC_ variants were the most common (4,253 out of 8,828), followed by *bla*_NDM_ (2,652 out of 8,828) and *bla*_OXA-48-like_ (2,429 out of 8,828).

A subset analysis of genomes from Indian clinical settings revealed a similar trend in virulence determinants but notable differences in resistance profiles. In this dataset, *iucA* was detected in 99.6% (523 out of 525) of genomes, while *rmpADC* was in 16% (87 out of 525), *rmpA2* was present in 36% (189 out of 525), *iroB* in 16% (84 out of 525) and *peg-344* in 38.09% (200 out of 525). However, the resistance landscape was as follows: 69.71% (366 out of 525) were ESBL producers carrying *bla*_CTX-M_, 76.95% (404 out of 525) of Indian genomes carried carbapenemase genes, predominantly *bla*_OXA-48-like_ (93.81%, 379 out of 404), followed by *bla*_NDM_ (10.89%, *n*=44/404), with some percentage among them having both the genes, and 4 isolates carrying *bla*_NDM_ co-carried *bla*_KPC_ gene and were detected.

Of the 525 Indian genomes analysed, 13.1% (69 out of 525) carried all 5 virulence genes. Among these, 15.9% (11 out of 69) also harboured carbapenemase genes, representing CR-hvKp. The remaining 81.2% (56 out of 69) lacked both ESBL and carbapenemase genes, suggesting that a majority of hypervirulent Kp in this setting circulate in non-ESBL, non-carbapenemase backgrounds.

### Primer design and *in silico* validation

We selected five candidate genes (*rmpA*, *rmpA2*, *iucA bla*_NDM_ and *bla*_OXA-48-like_) to differentiate CR-hvKp. These targets were selected based on their prevalence patterns observed in both global and Indian datasets. Primers for *iroB*, *peg-344* and *bla*_CTX-M_ were designed but not included in the primary panel, as these genes can be used in the panel on specific requirements. Similarly, *bla*_KPC_ is rarely found in Indian isolates; primers were included only for standardization purposes. All primers were designed or modified to target conserved regions of hypervirulence-associated genes (*rmpA*, *rmpA2*, *iucA*, *iroB* and *peg-344*) and resistance genes (*bla*_CTX-M_, *bla*_NDM_, *bla*_OXA-48-like_ and *bla*_KPC_ variants). Designed primers were optimized to have a G+C content near 50 mol%, a ΔTm<2 °C and a length of 18–22 bases. Evaluations using *in silico* PCR confirmed high specificity for target sequences and no significant off-target amplification. The designed primers generated *in silico* amplification products of *rmpA* (530 bp), *rmpA2* (470 bp) and *iucA* (170 bp), *iroB* (219 bp) and *peg-344* (313 bp) in hypervirulent reference strains such as *K. pneumoniae* KCTC 2242 (NC_017540) and *K. pneumoniae* NTUH-K2044 (NC_012731). The primers for *bla*_NDM_, *bla*_CTX-M_, *bla*_OXA-48-like_ and *bla*_KPC_ produced amplicons of 401, 560, 630 and 798 bp, respectively. The detailed list of all primers is mentioned in Table 1.

### Bacterial strain and phenotypic characterization

In this study, 150 clinical isolates Supplementary Material 1 (Table S4) of Kp associated with bacteraemia or respiratory infections were randomly selected for primer evaluation and phylogenomic analysis. Following confirmation of these isolates as Kp, they were assessed for hmv using the string test, which identified 22% (33 out of 150) as hmv-positive. Additionally, the isolates exhibited significant AMR, with only 30% (45 out of 150) showing susceptibility to carbapenems, specifically ertapenem and meropenem.

### Genome-based population structure of bacterial strains

The MST of the Kp study isolates reveals a diverse population structure, with clear clustering based on sequence types (STs). The predominant clone was ST2096, which comprised 26.7% (40 out of 150) isolates, indicating its dominance within the studied population. ST147, the second largest cluster with 21.3% (32 out of 150) isolates, is a globally recognized high-risk clone associated with carbapenem resistance. Another prominent cluster, ST231, associated with 13.3% of isolates, is notable for its strong association with AMR genes in Indian clinical settings. Hypervirulent clones, including ST23 and ST65, were observed in smaller clusters with 4% (6 out of 150) and 1.3% (2 out of 150) isolates, respectively. Additionally, the population demonstrates considerable genetic diversity, with smaller clusters of STs such as ST101, ST14, ST45 and ST16. The phylogenetic relationships inferred from the MST highlight the coexistence of MDR and hypervirulent clones, with evidence of convergence in certain lineages. Based on Kleborate, among the 150 clinical isolates, 105 were CRKp, and 45 were non-CRKp. Of the CRKp group, only two isolates (1.9%) carried all five virulence genes (*rmpA*, *rmpA2*, *iucA*, *iroB* and *peg-344*) and were classified as CR-hvKp. The majority, 79 isolates (75.2%), had less than 5 virulence genes (typically *iucA*) and were grouped under the virulence marker-positive cKp, while 24 (22.9 %) had no virulence genes. Among the 45 non-CRKp isolates, 22 (48.9%) were classified as hvKp (all 5 virulence genes), 12 (26.7 %) as cKp with partial virulence and 11 (24.4%) lacked all virulence genes Supplementary Material 2 (Table S1).

**Fig. 1.**
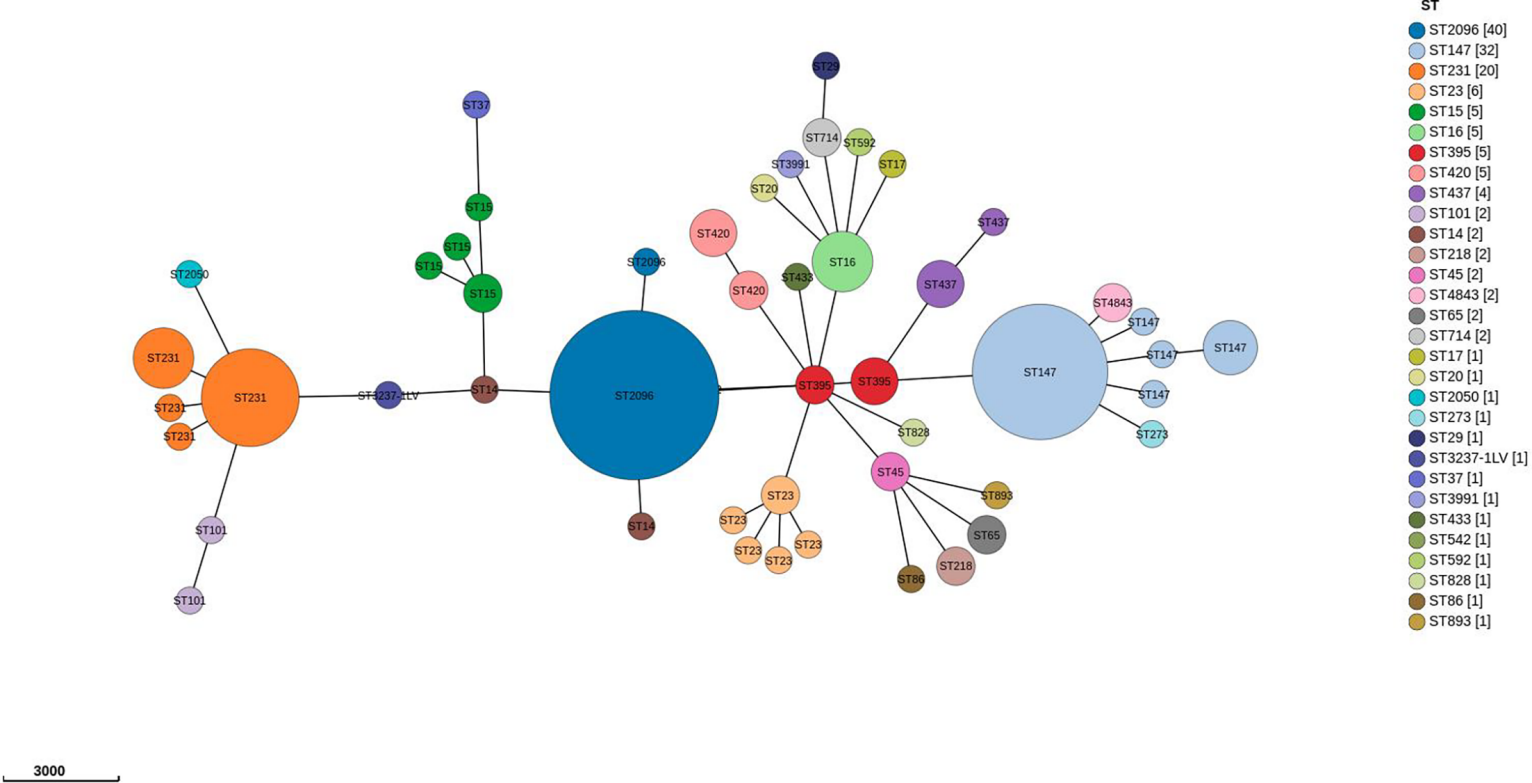
MST for *n*=150 isolates based on cgMLST. The tree was generated using GrapeTree, with nodes representing different STs. Node sizes are proportional to the number of isolates within each ST, and colours differentiate STs. The scale represents the genetic distance.

The MST based on cgMLST revealed the clonal distribution of Kp isolates. The predominant STs were ST2096 (40 isolates), ST147 (32 isolates) and ST231 (20 isolates), forming major clusters that were centrally positioned, linking multiple other STs and suggesting their epidemiological significance. Other notable STs included ST23, ST15, ST16, ST395 and ST420 (5–6 isolates each) (Fig. 1).

### Optimization of PCR conditions

The m-PCR assay was optimized to detect five target genes (*iucA*, *bla_NDM_*, *rmpA*, *rmpA2* and *bla*_OXA-48-like_) through a stepwise approach, progressing from a duplex to a pentaplex assay. The optimal annealing temperature was determined to be 50 °C, with 35 cycles ensuring successful amplification. This condition provided consistent and reliable results for all target genes when multiplexed Supplementary Material 2 (Fig. S3). For all the optimization experiments, positive control B1644 (accession ID: GCA_047922575.1) was used.

**Fig. 2.**
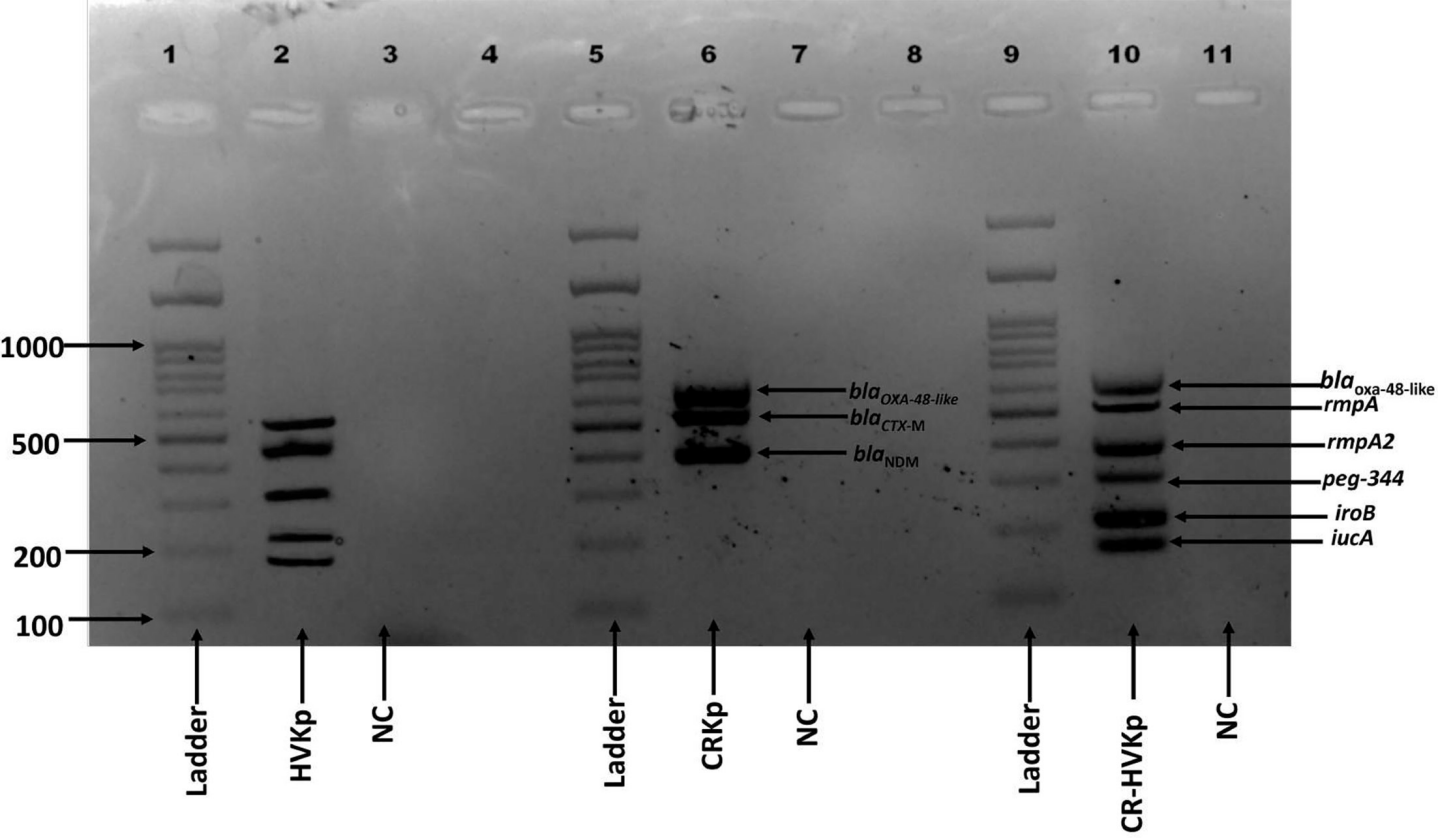
m-PCR detection of resistance and virulence genes in Kp. Lanes show amplification of *rmpA* (530 bp), *rmpA2 *(470 bp), *iucA* (170 bp), *iroB *(219 bp), *peg-344* (313 bp), *bla*_NDM_ (401 bp), *bla*_OXA-48-like_ (630 bp) and *bla*_CTX-M-15_ (560 bp) genes in 1 single Kp isolate, which is MDR-hvKp. A 100 bp DNA ladder is used. The presence of multiple bands indicates convergence of resistance and virulence traits.

Additionally, markers such as *iroB*, *peg-344* and *bla*_KPC_ were optimized as an extended version of the multiplex assay. The detailed cyclic conditions for all the markers are mentioned in Table 2*.* All the target genes were also amplified in singleplex PCR, confirming the functionality of individual primers. The m-PCR assay also demonstrated successful amplification of the selected markers shown in Fig. S4 (Supplementary Material 2). Agarose gel electrophoresis confirmed the successful amplification of target genes in the m-PCR assay. Various combinations of genes were tested to ensure optimal amplification without cross-reactivity (Fig. S5). Distinct bands correspond to *iucA*, *rmpA*, *rmpA2*, *iucA*, *peg-344*, *bla*_NDM_, *bla*_OXA-48-like_ and *bla*_CTX-M-15_, all observed in a single MDR-hvKp clinical isolate (ref ID:GCA_047922635.1), validating the specificity of the assay as observed in Fig. 2.

**Table 2.** Cycling conditions (Qiagen Multiplex)

	Temp. (°C)	Time	Cycles
**Initial denaturation**	95	10 min	35
**Denaturation**	94	30 sec	
**Annealing**	50	1	
**Extension**	72	1 min	
**Final extension**	72	10 min	
**Hold**	4	∞	

Table 1: Cycling conditions for the detection of virulence (*rmpA*, *rmpA2*, *iucA*, *iroB *and *peg-344*) and resistance (*bla*_NDM_, *bla*_OXA-48-like_ and *bla*_KPC_) genes, including annealing temperatures, cycle numbers and extension times for both the primary multiplex assay and the extended panel.

### Sensitivity and specificity

The assay demonstrated 100% specificity, with no amplification observed in non-target bacterial strains. Sensitivity tests revealed a consistent detection limit of 1 ng µl^−1^ for the target genes across triplicate experiments Supplementary Material 2 (Figs S1 and S2). Amplification results confirmed the presence of virulence genes (*rmpA*, *rmpA2* and *iucA*) and resistance genes (*bla*_NDM_ and *bla*_OXA-48-like_), with successful detection up to a bacterial load corresponding to a 10⁻² dilution (525 c.f.u. ml^−1^). For standardization, positive control B1644 (accession ID: GCA_047922575.1) was used.

To assess the feasibility of detecting multiple targets in a single reaction, we tested different gene combinations in m-PCR assays. Various sets of hypervirulence (*rmpA*, *rmpA2*, *iucA*, *iroB* and *peg-344*) and resistance markers (*bla*_NDM_, *bla*_OXA-48-like_, *bla_KPC_* and *bla*_CTX-M_) were evaluated to determine compatibility and amplification efficiency. The optimized pentaplex assay (*iucA*, *bla*_NDM_, *rmpA*, *rmpA2* and *bla*_OXA-48-like_) consistently yielded distinct and reproducible bands. Additional genes, including *iroB*, *peg-344*, *bla*_KPC_ and *bla*_CTX-M_, were incorporated in extended multiplex reactions, demonstrating successful amplification without cross-reactivity. These results confirm the adaptability of the assay for targeted applications, allowing for tailored detection based on specific diagnostic needs, as shown in Fig. S6 (Supplementary Material 2) . The *bla*_CTX-M_, although not included in the primary multiplex panel, was standardized using the same cycling conditions and showed amplification when run alongside virulence markers, as shown in Fig. S7 (Supplementary Material 2).

### Phenotypic and PCR correlation of hypervirulence and carbapenem resistance

The correlation between the hmv phenotype and virulence-associated markers was evaluated to assess the diagnostic accuracy of the string test. Among 150 isolates, the string test identified hmv in 33, of which 79% (26 out of 33) carried at least 1 virulence-associated gene (*rmpA*, *rmpA2* and *iucA*). However, 76% (88 out of 116) of hmv-negative isolates also harboured 1 or more of these markers. These findings indicate that the string test does not reliably identify hvkp or correlate with any pathotype as per the hvkp definition. In contrast, resistance phenotypes showed perfect correlation with genotypic findings: all 105 phenotypically carbapenem-resistant isolates carried either *bla*_OXA-48-like_ (59 out of 105), *bla*_NDM_ (21 out of 105) or both genes (25 out of 105), while none of the carbapenem-susceptible strains tested positive for these genes by PCR.

### WGS and PCR correlation of hypervirulence and carbapenem resistance

WGS and m-PCR analysis demonstrated a 100% correlation in detecting hypervirulence and carbapenem resistance determinants. All isolates carrying key virulence genes (*iucA*, *rmpA*, *rmpA2*, *iroB *and *peg-344*) in WGS were also confirmed by PCR, underscoring the reliability of the selected molecular markers. Similarly, carbapenem-resistant isolates harboured *bla*_OXA-48-like_, *bla*_NDM_ or both, with perfect concordance between WGS and PCR results. No discrepancies were observed, indicating that the developed m-PCR-based screening effectively detects hypervirulence and resistance markers with the same accuracy as WGS.

## Discussion

Healthcare-associated infections caused by Kp remain a critical global challenge due to their high prevalence, widespread AMR and diverse virulence characteristics [1,9]. The increasing incidence of CRKp strains has further complicated this issue, as many of these strains carry virulence-associated genes (*rmpA*, *rmpA2*, *iucA*, *iroB* and *peg-344*) alongside resistance determinants, resulting in severe and often untreatable infections [15,34]. Complicating their detection, many of these convergent clones, which exhibit both multidrug resistance and hypervirulence traits, lack the hmv phenotype traditionally assessed using the string test. This limitation significantly undermines the utility of phenotypic methods in accurately identifying these high-risk strains. To address these diagnostic challenges, we have attempted to systematically screen global hvKp genomes and developed a robust m-PCR assay to accurately identify CR-hvKp, addressing key gaps in existing diagnostic approaches.

Genome analysis of ~12,000 hvKp genomes from the NCBI Pathogen Detection Database revealed a high prevalence of hypervirulence-associated genes, with *iucA* (aerobactin) being the most frequently detected, followed by *peg-344/rmpA2*. While less common, other hypervirulence markers such as *iroB* and *rmpA* may also be useful depending on their regional distribution. Carbapenemase genes were identified in 68.7% (8,829 out of 12,846) of the genomes, with *bla*_KPC_ being the most prevalent globally. However, analysis of Indian clinical isolates demonstrated a predominance of *bla*_OXA-48-like_ genes, followed by *bla*_NDM_, highlighting significant regional differences in resistance mechanisms. These findings are consistent with previous reports indicating that *bla*_KPC_ is the dominant carbapenemase globally, whereas carbapenem resistance in Kp in India is often associated with endemic clones harbouring *bla*_OXA-48-like_ and *bla*_NDM_ [35,37]. Together, these genes serve as critical markers for both virulence and drug resistance, enabling comprehensive pathotype classification of clinical isolates. MST analysis of our 150 clinical isolates identified distinct clones circulating in India, including ST2096, ST147 and ST231, which differ from the globally dominant hospital-associated clones. Notably, 58% (87 out of 150) of the isolates were MDR Kp strains carrying at least 1 hypervirulence-associated gene, reflecting a substantial burden of strains with partial virulence potential. This high prevalence of MDR-Kp strains carrying one or more virulence markers underscores the clinical relevance of the m-PCR assay in the Indian context. Given the regional differences in clone distribution and resistome profiles, we have selected nine key genetic markers that serve as critical indicators of both virulence and drug resistance, enabling comprehensive pathotype classification of clinical isolates.

The significance of this assay lies in its ability to simultaneously detect key virulence and resistance determinants within a single reaction, thereby enhancing diagnostic efficiency. By eliminating the need for sequential testing or the resource-intensive process of WGS, this approach significantly reduces turnaround time while ensuring rapid and accurate pathogen characterization. Among the 150 clinical isolates tested, the assay demonstrated 100% concordance with WGS for *iucA*, *rmpA*, *rmpA2*, *bla*_NDM_ and *bla*_OXA-48-like_, with a detection sensitivity of 1 ng µl^−1^ DNA or 525 c.f.u. ml^−1^. This performance far exceeds that of the string test, which identified hmv in only 33 of 150 isolates, failing to detect another 88 isolates that harboured virulence markers. These findings underscore the limitations of phenotypic screening for hypervirulence. Such limitations, consistent with the findings of Russo *et al.* [23] and Zhu *et al.* [24], emphasize the unreliability of phenotypic tests for hvKp detection, particularly in cases where mutations in *rmpA/rmpA2* or alternative virulence factors like *peg-344* drive pathogenicity [27,38]. In contrast, all 105 carbapenem-resistant isolates exhibited perfect phenotypic–genotypic correlation, reaffirming the assay’s utility for CRKp detection. The ability to rapidly and accurately identify CR-hvKp could significantly reduce mortality rates, which often exceed 50% in delayed-treatment cases [15], and enhance antimicrobial stewardship, particularly in high-prevalence settings such as India, where 57% of Kp bloodstream infections are carbapenem-resistant [10].

Previous studies, such as those by Yu *et al.*, have attempted to differentiate carbapenem-resistant from hypervirulent Kp strains using m-PCR assays [39]. While they developed three sets of multiplex primers wherein the first set of primers was designed specifically for certain STs (ST11/258, ST23, ST86, ST65 and ST375), the second set was for capsular polymerase genes specific to various K types like K1, K2, KL64 and KL47 [39]. Similarly, another study developed an m-PCR assay targeting seven virulence genes (*rmpA*, *allS*, *kfu*, *iuc*, *iro*, *fimH* and *uge*) along with K1/K2 capsular serotypes, enhancing the detection of hypervirulent strains [40]. More recently, LAMP and multiplex qRT-PCR assays offer improved sensitivity and specificity for detecting hypervirulent Kp [41]. While these assays contribute to rapid and specific identification, the m-PCR approach is confined to predefined virulence genes and capsular types, potentially overlooking emerging genetic variants and converging traits. A previous study from our setting [26] prioritized *iucA* as the sole marker for hvKp detection. However, our inclusion of multiple virulence markers addresses variability in *rmpA/rmpA2*, as highlighted by Lin *et al.* [27]. Our assay, in contrast, provides a more comprehensive solution by simultaneously detecting both virulence and resistance factors, overcoming the limitation of focusing on a narrow set of markers. Our assay is designed to work effectively within the Indian subcontinent’s genomic diversity, including prevalent STs like ST2096, ST147 and ST231, which are not commonly identified in studies from regions such as the USA. This region-specific focus is essential for the accurate diagnosis of Kp infections and underscores the importance of tailored diagnostic tools.

A key strength of our m-PCR assay is its comprehensive detection capability, enabling the simultaneous identification of all three major Kp pathotypes (CRKp, hvKp and CR-hvKp) along with the virulence marker-positive cKp. Unlike the string test, which is subjective and prone to false negatives, this molecular approach accurately detects both virulence and resistance determinants, ensuring earlier diagnosis and intervention critical for preventing severe hvKp infections. Additionally, the assay’s high specificity ensures that only Kp genes are amplified, eliminating the risk of cross-reactivity with other bacterial species. To further enhance its clinical utility, we have incorporated regionally relevant targets, optimizing five core genes (*iucA*, *rmpA*, *rmpA2*, *bla*_NDM_ and *bla*_OXA-48-like_) while also allowing for the inclusion of *peg-344*, *iroB*, *bla*_KPC_ and *bla*_CTX-M_ based on epidemiological needs. These adaptations improve the global applicability of the assay. To better scope and contextualize the performance of our m-PCR assay, we compared it with several recently published assays in Table S2 Supplementary Material 2 [42,43]. Compared to WGS and singleplex PCR, which are expensive and time-consuming, this m-PCR assay offers a cost-effective, high-throughput solution suitable for routine diagnostics, particularly in resource-limited settings. Since this assay has been validated on diverse clinical isolates across India, it offers a high-throughput, reliable alternative to traditional diagnostic methods, making it especially valuable for clinical diagnostics in Indian hospitals and similar global settings. Despite these strengths, there are limitations to the m-PCR assay. We have demonstrated strong concordance with WGS-based data; we did not conduct a head-to-head comparison with other amplification platforms such as qPCR or LAMP; this remains a valuable avenue for future benchmarking. While *bla*_CTX-M_ was not multiplexed with the core five-gene panel, it was successfully optimized under the same cycling conditions and can be included separately. This design preserves assay flexibility and enables regional customization based on resistance profiles. Our current validation cohort was cross-sectional, and the performance of this assay in longitudinal surveillance or outbreak settings remains an important future direction to assess its real-time epidemiological utility. Despite the fact that the isolates originated from distinct patients, we cannot completely exclude the possibility that clonal spread could occur within the same ward or hospital. This could help clarify the significant genetic similarity noted and should be taken into account when analysing prevalence and the distribution of resistance and virulence. While the assay targets the most informative virulence and resistance genes relevant to CR-hvKp detection, additional modules such as capsular typing (K-locus) and virulence genes associated with biofilm formation could be explored to expand diagnostic depth.

In conclusion, this study presents the development and validation of an m-PCR assay that offers a significant improvement over existing methods for detecting Kp pathotypes in clinical isolates. By simultaneously detecting virulence and resistance genes, the assay provides a comprehensive diagnostic tool that is suitable for rapid clinical implementation. Beyond clinical diagnostics, this assay enhances molecular surveillance and outbreak investigations by enabling real-time tracking of strain prevalence. Its ability to rapidly and accurately identify carbapenem-resistant and hypervirulent Kp supports infection control efforts, targeted interventions and antimicrobial stewardship in both healthcare and community settings. Overall, this assay provides a valuable solution for managing Kp infections in India and similar high-burden regions, addressing the growing challenge of AMR and hypervirulence through efficient and accessible molecular diagnostics.

## Supplementary material

10.1099/jmm.0.002090 Supplementary Material 1.

10.1099/jmm.0.002090 Supplementary Material 2.

## Abbreviations

AMR: antimicrobial resistance
cgMLST: core genome MLST
ESBL: extended-spectrum *β*-lactamase
hmv: hypermucoviscous
Kp: *Klebsiella pneumoniae*
MDR: multidrug-resistant
m-PCR: multiplex PCR
MSTs: minimum spanning trees
STs: sequence types
WGS: whole-genome sequencing
