# Supplementary material for: Multiplex PCR assay for the rapid detection of Klebsiella pneumoniae pathotypes
Source: J Med Microbiol. 2025 Oct 31;74(10):002090. doi: 10.1099/jmm.0.002090 (PMC12578128; doi:10.1099/jmm.0.002090)
Supplement: Supplementary Material 2. [file jmm-74-02090-s002.pdf]

Supplementary Figures

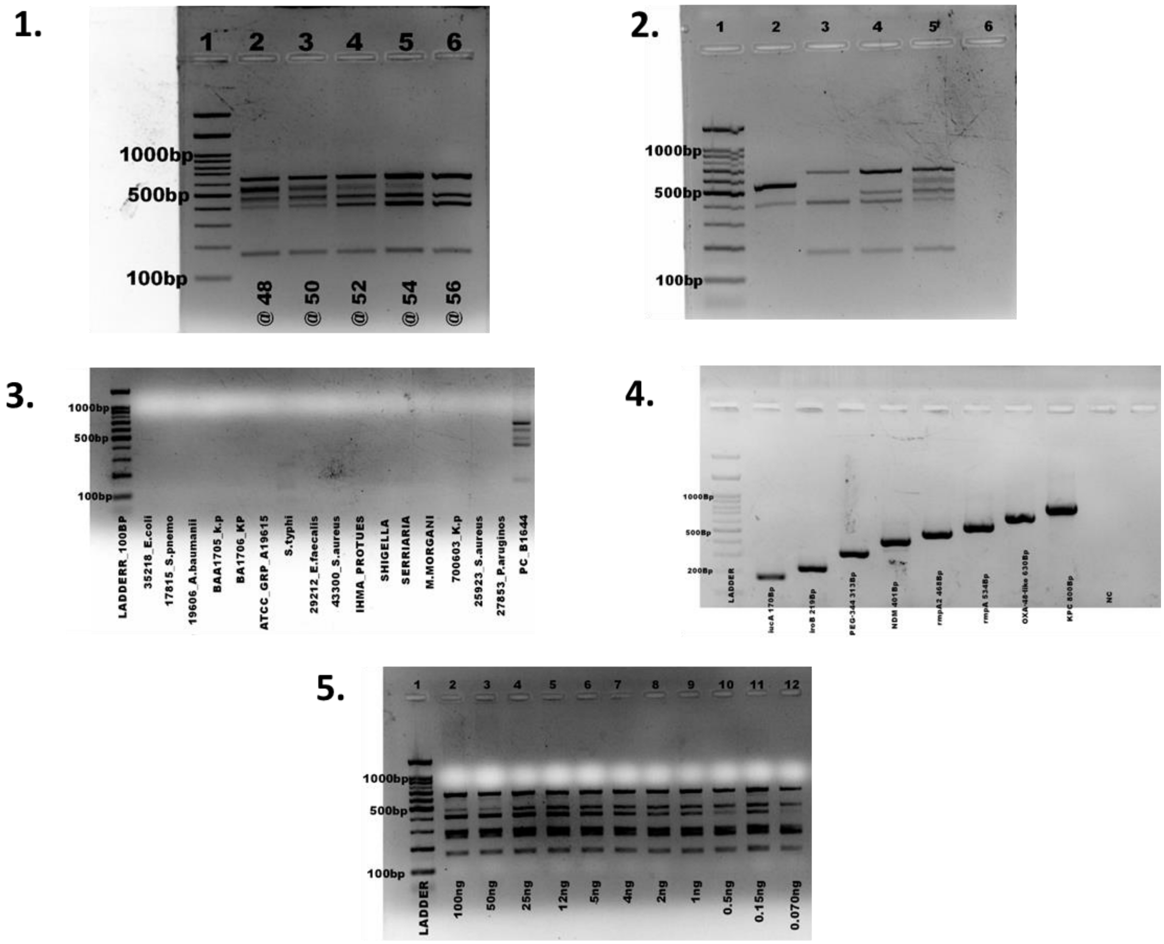

Supplementary Figure 1. Determination of annealing temperature of the multiplex PCR. Lane 1: 1000 bp DNA marker; Lane 1: 48°C annealing; Lane 2: 50°C annealing; Lane 3: 52°C annealing; Lane 4: 54°C annealing; Lane 5:56°C annealing. **Figure 2.** Optimization of the primers of m-PCR. Lane 1: 1000 bp DNA marker; Lane 1: duplex PCR; Lane 2: triplex PCR; Lane 3: quadruplex PCR; Lane 4: pentaplex PCR formed by *iucA(170bp)*, *bla*$_{NDM}$ *(401bp)*, *rmpA (534bp)*, *rmpA2(468bp)*, and *bla*$_{OXA\text{-}4\text{-}like}$ *(630bp)*, **Figure 3**. Determination of the specificity of the multiplex PCR. Lane 1: 1000 bp DNA marker, Lane 2-16: the template 14 bacterial strains of m-PCR, which are negative. Lane 17: positive control. **Figure 4.** Amplification of the target gene of the multiplex PCR. Lane 1: 1000 bp DNA marker; Lanes 2–9: the template of single genes amplification, respectively for *iucA(170bp)*, *iroB(219bp)*, *peg-344(313bp)*, *bla*$_{NDM}$*(401bp)*, *rmpA2(468bp)*, *rmpA(534bp)*, *bla*$_{OXA\text{-}48\text{-}like}$*(630bp)*, *and bla*$_{KPC}$ *(798bp)*. **Figure 5**. Determination of the sensitivity of the multiplex PCR for bacterial genomic DNA detection for strain **GCA_047922575.1**, Lane 1: 1000 bp DNA marker; a Lanes 2–12: the concentration of Kp DNA were 100 ng, 50 ng, 25 ng, 12 ng, 5 ng, 4 ng, 1 ng, 500 pg, 150 pg, and 70 pg, respectively.

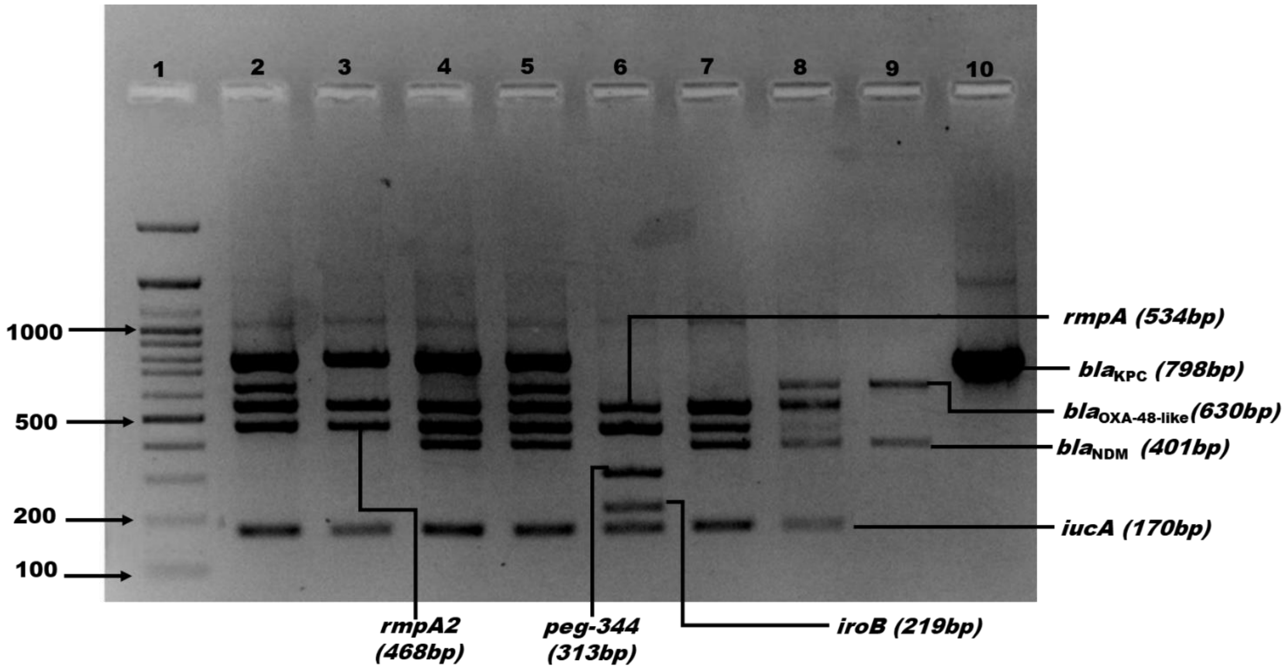

Supplementary Figure 6. We evaluated various multiplex PCR combinations to determine the optimal setup for detecting key virulence [*rmpA(534bp), rmpA2(468bp), iucA(170bp), iroB(219bp), peg-344(313bp)*]and resistance [bla_{NDM}*(401bp),* $bla_{OXA\text{-}48\text{-}\ like}$ *(630bp),* bla_{KPC}*(798bp)*] genes. Different gene combinations were tested to assess their compatibility within a single assay. Lane-wise, the results are as follows: Lane 1 contains the molecular size marker (ladder), followed by multiplex amplification of *rmpA(534bp), rmpA2(468bp), iucA(170bp),* $bla_{OXA\text{-}48\text{-}like}$ *(630bp),* bla_{KPC} *(798bp),* (Lane 2), *rmpA, rmpA2, iucA,* bla_{KPC}*,* bla_{NDM} (Lane 3), and *rmpA, rmpA2, iucA,* bla_{KPC}*,* bla_{NDM}*,* $bla_{OXA\text{-}48\text{-}like}$ (Lane 4). Lane 5 includes the extended panel with *rmpA, rmpA2, iucA, iroB, and peg-344*, while Lane 6 and Lane 7 represent multiplex combinations with *rmpA, rmpA2, iucA,* bla_{NDM} and *rmpA, rmpA2, iucA,* bla_{NDM}*,* $bla_{OXA\text{-}48\text{-}like}$, respectively. Lane 8 shows amplification of $bla_{OXA\text{-}48\text{-}like}$ and bla_{NDM} alone, whereas Lane 9 confirms the presence of bla_{KPC} individually. These results highlight the feasibility of a robust multiplex PCR approach for detecting hypervirulence and carbapenem resistance markers in Kp , with additional genes available for extended analysis as needed.

7.

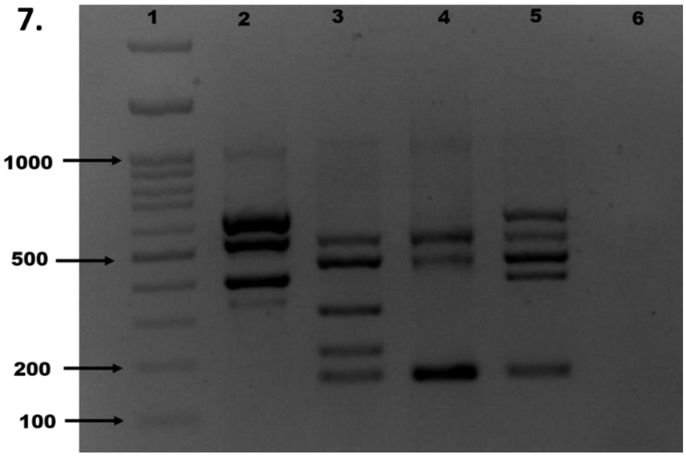

Supplementary Figure 7. m-PCR amplification of resistance and virulence genes; addition and standardization with *bla*$_{\text{CTX-M}}$. Lane 1: 100 bp DNA ladder, Lane 2: *bla*$_{\text{OXA-48-like}}$ (630bp), *bla*$_{\text{CTX-M}}$ (560bp), *bla*$_{\text{NDM}}$ (401bp), Lane 3: Full virulence genes panel – *rmpA, rmpA2, iucA, iroB, peg-344*, Lane 4: Partial virulence panel – *rmpA*(534bp) *, rmpA2, iucA*, Lane 5: Combined multiplex panel – simultaneous amplification of *rmpA, rmpA2, iucA*, *bla*$_{\text{OXA-48-like}}$, and *bla*$_{\text{NDM}}$, Lane 6: Negative control

Supplementary Table 1: Distribution of the n=150 Kp isolates

Category	Definition	Number of Isolates (n)	Percentage (%)
**CR-hvKp**	CRKp isolates with all 5 virulence genes (*rmpA, rmpA2, iucA, iroB, peg-344*)	2	1.3%
**CRKp with partial virulence**	CRKp isolates with fewer than 5 virulence genes (*typically only iucA*)	79	52.7%
**CRKp**	CRKp isolates lacking all 5 virulence markers	24	16.0%
**hvKp (non-CR)**	Non-CRKp isolates carrying all 5 virulence genes	22	14.7%
**CRKp (virulence-marker positive)**	CRKp isolates with <5 virulence genes	12	8.0%
**cKp (non-CR, non-virulent)**	Isolates lacking both carbapenem resistance genes and all 5 virulence genes	11	7.3%
**Total**		150	100%

Supplementary Table 2: Comparative Summary of Existing Diagnostic Assays for hvKp and CRKp

Sr no.	Detection Method	Gene Targets	Multiplexed	All Genes Detected Simultaneously	Sensitivity / Specificity	Turnaround Time	Reference
1	Conventional PCR	*rmpA, rmpA2, iucA, iroB, peg-344* + resistance genes	Yes	Yes	Not reported in % terms	~3–4 hrs	(37)
2	Recombinase-Aided Amplification (RAA)	*rmpA, iucA, peg-344*	Run separately	No	100% / 96.7%	~15–30 min	(25)
3	LAMP	bla_{KPC}, bla_{NDM}, bla_{OXA-48}, bla_{VIM}	Run separately	No	>95% / >95% (varies per gene)	~30–45 min	(41)
4	qRT-PCR	*rmpA, rmpA2, iucA,* bla_{KPC}	Yes	Yes	97.1% / 100%	~2–2.5 hrs	(42)
5	LAMP + qRT-PCR	*rmpA, iucA, peg-344*	(qRT-PCR) / (LAMP)	Yes (qRT-PCR)	100% / 97.9% (qRT-PCR)	~30 min (LAMP), ~2 hrs (qRT-PCR)	(39)
6	Multiplex Conventional PCR	*rmpA, rmpA2, iucA, iroB, peg-344,* $bla_{OXA-48-like}$, bla_{NDM}	Yes	Yes	98.6% / 100% (vs. WGS)	~3 hrs	Present study
